# Obstetric and neonatal outcomes following taxane use during pregnancy: a systematic review

**DOI:** 10.1186/s12885-023-11704-6

**Published:** 2024-01-02

**Authors:** Alejandro Aranda-Gutierrez, Ana S. Ferrigno Guajardo, Bryan F. Vaca-Cartagena, David G. Gonzalez-Sanchez, Arantxa Ramirez-Cisneros, Andrea Becerril-Gaitan, Hatem A. Azim, Cynthia Villarreal-Garza

**Affiliations:** 1 Department of Hemato-Oncology, Instituto Nacional de Ciencias Medicas y Nutricion Salvador Zubiran, Mexico City, Mexico; 2 Department of Medicine, Yale University School of Medicine, New Haven, CT USA; 3 Breast Cancer Center, Hospital Zambrano Hellion, Tecnologico de Monterrey, San Pedro Garza Garcia, Mexico

**Keywords:** Cancer, Pregnancy, Chemotherapy, Taxanes, Neonatal outcomes, Obstetric outcomes

## Abstract

**Background:**

The use of taxanes following the first trimester of pregnancy is endorsed by current clinical guidelines. However, evidence regarding their safety in terms of obstetric and neonatal outcomes is limited.

**Methods:**

A comprehensive literature search was performed using the MEDLINE, CENTRAL and Web of Sciences databases from their inception up to 12/16/2022. Eligibility criteria included gestational taxane use, presentation of original findings, and individual case data presented. A descriptive statistical analysis was undertaken.

**Results:**

A total of 159 patients treated with taxane-containing regimens during pregnancy were identified, resulting in 162 fetuses exposed in utero. The majority of patients had breast cancer (*n* = 88; 55.3%) or cervical cancer (*n* = 45; 28.3%). The most commonly employed taxane was paclitaxel (*n* = 131; 82.4%). A total of 111 (69.8%) patients were also treated with other cytotoxic drugs during pregnancy, including platinum salts (*n* = 70; 63.0%) and doxorubicin/cyclophosphamide (*n* = 20; 18.0%). While most patients received taxanes during the second trimester of pregnancy (*n* = 79; 70.0%), two were exposed to taxanes in the first trimester.

Obstetric outcomes were reported in 105 (66.0%) cases, with the most frequent adverse events being preterm contractions or premature rupture of membranes (*n* = 12; 11.4%), pre-eclampsia/HELLP syndrome (*n* = 6; 5.7%), and oligohydramnios/anhydramnios (*n* = 6; 5.7%). All cases with pregnancy outcome available resulted in live births (*n* = 132). Overall, 72 (54.5%) neonates were delivered preterm, 40 (30.3%) were classified as small for gestational age (SGA), and 2 (1.5%) had an Apgar score of < 7 at 5 min. Perinatal complications included acute respiratory distress syndrome (*n* = 14; 10.6%), hyperbilirubinemia (*n* = 5; 3.8%), and hypoglycemia (*n* = 2; 1.5%). In addition, 7 (5.3%) cases of congenital malformations were reported. At a median follow-up of 16 months, offspring health status was available for 86 (65.2%), of which 13 (15.1%) had a documented complication, including delayed speech development, recurrent otitis media, and acute myeloid leukemia.

**Conclusions:**

Taxanes appear to be safe following the first trimester of pregnancy, with obstetric and fetal outcomes being similar to those observed in the general obstetric population. Future studies should aim to determine the most effective taxane regimen and dosage for use during gestation, with a specific focus on treatment safety.

## Introduction

The simultaneous diagnosis of cancer and pregnancy is a rare clinical occurrence, complicating only 0.03–0.1% of gestations [1]. Despite its infrequency, it is imperative that obstetricians, oncologists, and surgeons possess a thorough understanding of its management as over 100,000 cases are reported annually and its incidence is expected to rise as a result of delayed childbearing and a higher proportion of affected patients choosing active oncologic treatment over pregnancy termination [2]. The treatment of these patients necessitates consideration of various factors, including the type and stage of cancer, gestational age, and the preferences of expectant parents. Moreover, the decision-making process must weigh maternal and fetal outcomes while carefully balancing the risks and benefits of each treatment modality. In particular, cytotoxic systemic treatments pose a challenging medical conundrum as the antineoplastic effects must be carefully balanced against the potential mutagenic, teratogenic, and carcinogenetic effects on the placenta and the developing embryo [3].

Current National Comprehensive Cancer Network guidelines recommend the use of certain chemotherapy regimens such as anthracyclines and alkylating agents following the first trimester (up to 14 weeks of gestation) [4]. The use of taxane-containing regimens during pregnancy is also endorsed when clinically indicated by disease status, although this recommendation is mostly based on limited retrospective data. Previous studies have indicated that taxanes may elevate the incidence of particular complications such as preterm contractions, premature rupture of membranes (PROM), small for gestational age (SGA) neonates, and admission to the neonatal intensive care unit [5].

Given that the use of taxanes has demonstrated a survival benefit in multiple malignancies, including those most commonly diagnosed during gestation (i.e., breast, cervical, and ovarian cancers) [6], it is imperative for clinicians to possess up-to-date evidence regarding their safety profile during gestation.

## Methods

We conducted a comprehensive literature search using MEDLINE, CENTRAL and Web of Sciences databases from their inception up to December 16, 2022 using the following search strategy: (pregnan* OR gestation*) AND (cancer* OR carcinoma OR malignan* OR neoplasm*) AND (taxan* OR paclitaxel OR docetaxel OR taxol OR taxotere). Retrieved references were evaluated independently by two authors for eligibility. Studies about patients receiving taxanes during pregnancy and detailing fetal outcomes were included. Inclusion criteria were (1) articles written in English, Spanish, or French, (2) presentation of original findings (e.g. not reviews), (3) gestational taxane use, and (4) individual case data presented. In cases of potential overlap, we prioritized articles with the greater number of patients or detailed information. Additionally, we reviewed the references of eligible studies to minimize inadvertent exclusion of relevant literature.

Data from all eligible articles were extracted in duplicate by two authors, with any disagreements resolved by a third author. Data about the following variables were recorded when available: the number of cases involving gestational taxane use, type of malignancy, concurrent use of non-taxane chemotherapy regimens, disease stage at diagnosis, specific taxane employed, gestational trimester at taxane initiation, taxane dose and frequency of administration, adverse events reported during pregnancy, gestational age at delivery, neonatal birth weight, Apgar scores at 1 and 5 min, neonatal complications, presence of congenital malformations, and pediatric complications. Information regarding whether the neonate was assessed as small for gestational age (SGA) was either directly extracted from the included reports included (if mentioned in the text) or evaluated by the authors using The World Health Organization Fetal Growth Charts and the definition of weight below the 10^th^percentile for neonates of the same sex and gestational age [7].

Median follow-up for neonates’ health status after birth was calculated using individual case data.

A descriptive statistical analysis was carried out using the SPSS Statistics software (IBM Corp., Armonk, N.Y., USA).

## Results

**Fig. 1:**
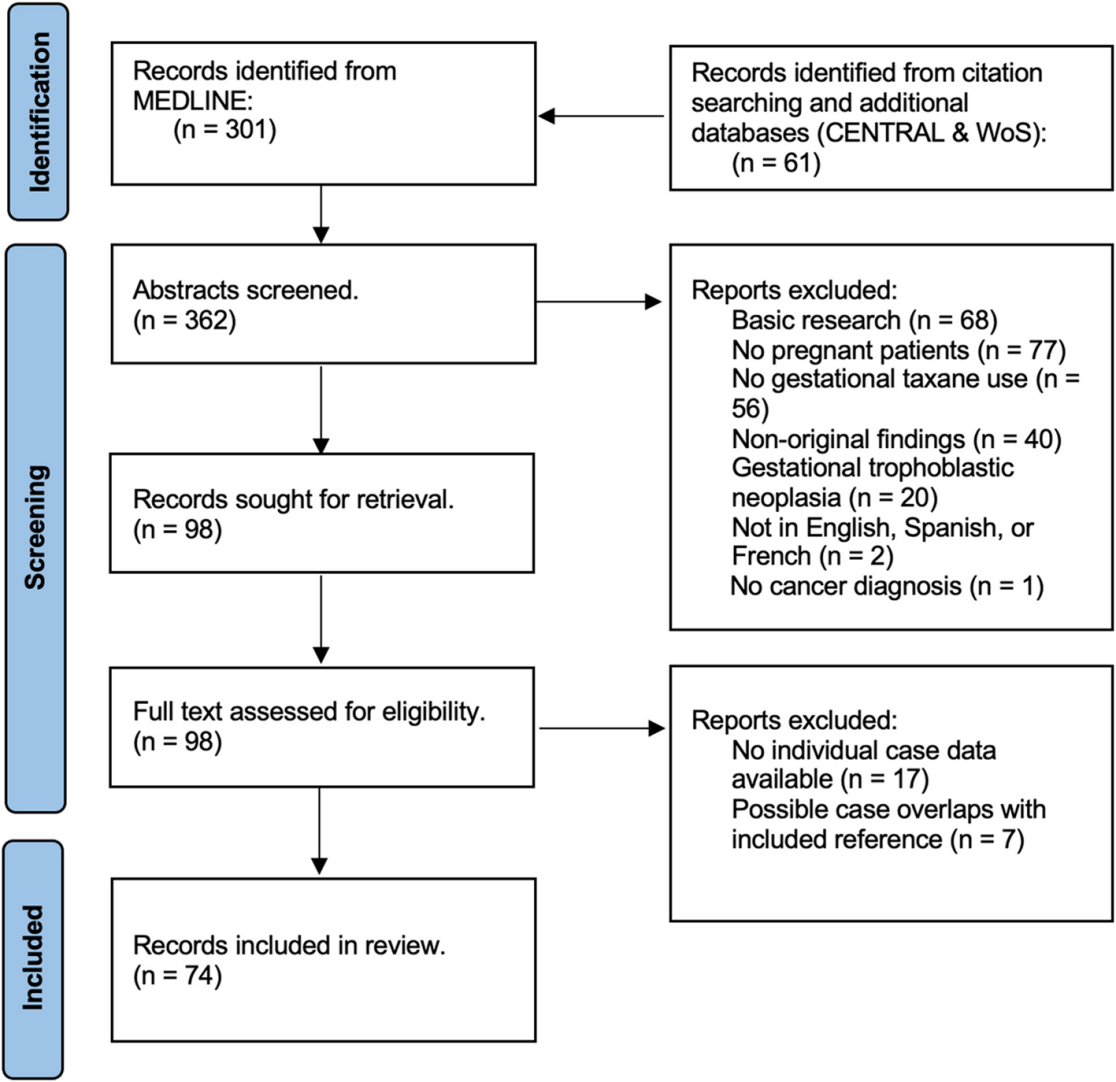
Flow diagram for process of reference selection after systematic search of literature

The search yielded a total of 362 unique records, of which 74 reports were included in the analysis (Fig. 1). Overall, 159 patients treated with taxane-containing regimens during pregnancy were identified in the literature, resulting in 162 fetuses exposed to taxanes in utero (including three twin pregnancies). Maternal and gestational data are summarized in Table 1. The median maternal age was 34 years (range: 22–44, *n* = 97). In most cases, the primary malignancy was diagnosed during pregnancy (*n* = 136; 93.2%), while a minority of cases involved disease recurrence (*n* = 8; 5.5%) or pregnancies detected while receiving chemotherapy (*n* = 2; 1.4%). Among those diagnosed during pregnancy, 55.1% were in the second trimester of pregnancy (*n* = 75), 25.0% in the first trimester (*n* = 34), 5.9% in the third trimester (*n* = 8), and undisclosed in 14.0% (*n* = 19). The median gestational age at diagnosis was 17 weeks (range 2 to 32; *n* = 105). Most patients were diagnosed with breast cancer (BC) (*n* = 88; 55.3%), followed by cervical (*n* = 45; 28.3%), ovarian (*n* = 18; 11.3%), lung (*n* = 6; 3.8%), oral squamous cell (*n* = 1; 0.6%), and gastric (*n* = 1; 0.6%) malignancies. Only 15 cases out of 123 (12.2%) that reported clinical stage had distant metastatic disease at diagnosis.

**Table 1:** Maternal data

	***n *(%)**
**Median maternal age at cancer diagnosis (*n* = 97)**	34 years (range 22–44)
**Median gestational age at cancer diagnosis (*n* = 105)**	17 weeks (range 2–32)
**Timing of cancer diagnosis (*n* = 146)**	
Primary tumor diagnosed during pregnancy	136 (93.2%)
Recurrent disease detected during pregnancy	8 (5.5%)
Detection of pregnancy during chemotherapy	2 (1.4%)
**Trimester at cancer diagnosis (*n* = 136)**	
First	34 (25.0%)
Second	75 (55.1%)
Third	8 (5.9%)
Not specified	19 (14.0%)
**Cancer type (*n* = 151)**	
Breast	88 (55.3%)
Cervical	45 (28.3%)
Ovarian	18 (11.3%)
Lung	6 (3.8%)
Oral squamous cell	1 (0.6%)
Gastric	1 (0.6%)
**Type of taxane administered during pregnancy (*n* = 159)**	
Paclitaxel	131 (82.4%)
Docetaxel	28 (17.6%)
**Gestational age at taxane initiation (*n* = 113)**	
First trimester	2 (1.8%)
Second trimester	79 (70.0%)
Third trimester	32 (28.3%)
**Other employed chemotherapy agents (*n* = 111)**	
Cisplatin	38 (34.2%)
Carboplatin	32 (28.8%)
Doxorubicin/cyclophosphamide	20 (18.0%)
Doxorubicin	6 (5.4%)
Epirubicin	5 (4.5%)
5-fluorouracil plus doxorubicin/cyclophosphamide	3 (2.7%)
Epirubicin/cyclophosphamide	2 (1.8%)
5-fluorouracil plus epirubicin/cyclophosphamide	1 (0.9%)
Cisplatin/gemcitabine	1 (0.9%)
Cyclophosphamide	1 (0.9%)
Vinorelbine	1 (0.9%)
Tegafur/gimeracil/oteracil	1 (0.9%)

The most commonly prescribed taxane regimen during pregnancy was paclitaxel (*n* = 131; 82.4%), followed by docetaxel (*n* = 28; 17.6%). In three cases (1.9%), the initial agent prescribed during pregnancy had to be switched to another taxane due to hypersensitivity reactions, resulting in gestational exposure to both paclitaxel and docetaxel. Regarding the gestational age at initiation of taxanes, most patients were treated in the second trimester (*n* = 79; 70.0%), while the remainder received them during the third (*n* = 32; 28.3%) and first trimesters (*n* = 2; 1.8%). Of note, the two patients that received taxanes during the first trimester of gestation had unrecognized pregnancies at the time.

The median gestational week at taxane initiation was 23.5 weeks (range 1 to 34; *n* = 91), occurring a median of 4 weeks (range 0–27; *n* = 76) after cancer diagnosis. In 73 patients for whom data was available, paclitaxel regimen dosages were 175 mg/m^2^ (*n* = 28; 38.4%), 135 mg/m^2^ (*n* = 19; 26.0%), and 80 mg/m^2^ (*n* = 15; 20.5%). For patients who received docetaxel (n = 11; 15.1%), the most frequently administered dose was 75 mg/m^2^ (*n* = 5; 45.5%). The median cumulative dose during pregnancy was 405 mg/m^2^ (range: 125–1200) for patients treated with paclitaxel and 320 mg/m^2^ (range: 160–650) for those prescribed docetaxel. In total, 111 patients (69.8%) also received other cytotoxic agents during pregnancy. These included cisplatin (*n* = 38; 34.2%), carboplatin (*n* = 32; 28.8%), doxorubicin/cyclophosphamide (*n* = 20; 18.0%), doxorubicin alone (*n* = 6; 5.4%), epirubicin alone (*n* = 5; 4.5%), 5-fluorouracil combined with either doxorubicin/cyclophosphamide (*n* = 3; 2.7%) or epirubicin/cyclophosphamide (*n* = 1; 0.9%), epirubicin/cyclophosphamide (*n* = 2; 1.8%), cisplatin/gemcitabine (*n* = 1; 0.9%), cyclophosphamide alone (*n* = 1; 0.9%), vinorelbine (*n* = 1; 0.9%), and tegafur/gimeracil/oteracil (*n* = 1; 0.9%). Additionally, 6 cases (3.8%) received trastuzumab, and 4 (2.5%) received granulocyte colony-stimulating factor.

Obstetric outcomes (Table 2) were reported in 105 (66.0%) cases. Common adverse events included preterm contractions or PROM (*n* = 12; 11.4%), pre-eclampsia/HELLP syndrome (*n* = 6; 5.7%), oligohydramnios/anhydramnios (*n* = 6; 5.7%), neutropenia (*n* = 6; 5.7%, with at least two cases classified as grade III/IV), anemia (*n* = 5; 4.8%, with at least one case classified as grade III/IV), thrombocytopenia (*n* = 4; 3.8%, with at least one case classified as grade III/IV), gestational diabetes (*n* = 3; 2.9%), and intrauterine growth restriction (*n* = 3; 2.9%). All cases of gestational taxane with pregnancy outcome available resulted in live births (*n* = 132). Neonatal outcomes are summarized in Table 3. Overall, 72 (54.5%) neonates were delivered preterm: 49 [68.1%] between 34 and 36 weeks and 6 days of gestation (late preterm), 16 [22.2%] between 32 and 33 weeks and 6 days of gestation (moderate preterm), 6 [8.3%] between 28 and 31 weeks and 6 days of gestation (very preterm), and 1 [1.4%] before 28 weeks of gestation (extremely preterm). In addition, 40 (30.3%) were classified as SGA and 2 (1.5%) had an Apgar score at 5 min < 7. Reported perinatal complications in the offspring exposed to taxanes in utero included acute respiratory distress syndrome (ARDS) (*n* = 14; 10.6%), hyperbilirubinemia (*n* = 5; 3.8%), hypoglycemia (*n* = 2; 1.5%), transient acute kidney injury (*n* = 1; 0.8%), mild hydrocephalus (*n* = 1; 0.8%), first grade intraventricular hemorrhage (*n* = 1; 0.8%), neutropenia (*n* = 1; 0.8%), and thrombocytopenia (*n* = 1; 0.8%). The distribution of these complications according to gestational age at birth was as follows: 5.7% of term neonates, 20.4% of late preterm neonates, 26.7% of moderate preterm neonates, 37.5% of very/extremely preterm neonates, and 85.7% of neonates of unknown gestational age. Specifically for ARDS, all affected neonates were born premature (42.9% late preterm, 14.3% moderate preterm, 21.4% very preterm, and 21.4% unknown). Out of 132 cases, 7 (5.3%) instances of congenital anomalies were reported: cleidocranial dysostosis [8], hip dysplasia (secondary to breech birth) [8], mitral valve stenosis [8], bilateral talipes equinovarus [9], pyloric stenosis [10], hypospadias [11], and multiple congenital malformations not otherwise specified [12].

**Table 2:** Obstetric outcomes

	***n*** **(%)**
Preterm contractions or premature rupture of membranes	12 (11.4%)
Pre-eclampsia/HELLP syndrome	6 (5.7%)
Oligohydramnios/anhydramnios	6 (5.7%)
Neutropenia	6 (5.7%)
Anemia	5 (4.8%)
Thrombocytopenia	4 (3.8%)
Gestational diabetes	3 (2.9%)
Intrauterine growth restriction	3 (2.9%)

**Table 3:** Neonatal and perinatal outcomes

	***n*** **(%)**
Preterm (< 37 weeks of gestation)	72 (54.5%)
Small for gestational age	40 (30.3%)
Apgar score at 5 min < 7	2 (1.5%)
Acute respiratory distress syndrome	14 (10.6%)
Hyperbilirubinemia	5 (3.8%)
Hypoglycemia	2 (1.5%)
Transient acute kidney injury	1 (0.8%)
Mild hydrocephalus	1 (0.8%)
First grade intraventricular hemorrhage	1 (0.8%)
Neutropenia	1 (0.8%)
Thrombocytopenia	1 (0.8%)
Cleidocranial dysostosis	1 (0.8%)
Hip dysplasia (secondary to breech birth)	1 (0.8%)
Mitral valve stenosis	1 (0.8%)
Bilateral talipes equinovarus	1 (0.8%)
Pyloric stenosis	1 (0.8%)
Hypospadias	1 (0.8%)
Multiple congenital malformations	1 (0.8%)

Information of health status after birth was available for 86 (65.2%) (median follow-up 16 months, range 1 to 160), of which 80% had a follow-up of ≥ 1 year. Overall, a complication was reported in 13 (15.1%) of the offspring and consisted of delayed speech development (*n* = 2; 2.0%), recurrent otitis media (*n* = 2; 2.0%), acute myeloid leukemia (*n* = 2; 2.0%), growth restriction (i.e., < 5% height and weight for age, *n* = 1; 1.0%), retroperitoneal embryonal rhabdomyosarcoma (*n* = 1; 1.0%), bilateral hearing loss (this offspring was also exposed in utero to cisplatin, *n* = 1; 1.0%), eczema (*n* = 1; 1.0%), iron deficiency anemia (*n* = 1; 1.0%), IgA deficiency (*n* = 1; 1.0%), autism spectrum disorder (*n* = 1; 1.0%), and a not otherwise specified life-threatening event at 3 months of age (*n* = 1; 1.0%).

## Discussion

Previous studies have investigated the safety of taxane use during pregnancy. Mir et al. conducted a systematic review regarding the safety of taxanes during gestation and found a favorable toxicity profile after examining the outcomes of 40 cases of pregnancy-associated BC [13]. Similarly, systematic reviews by Zagouri et al. have demonstrated a low prevalence of maternal and perinatal complications after treatment with taxanes during pregnancy in patients with BC (50 cases), cervical cancer (14 cases), and ovarian cancer (11 cases) [14–16]. In a European registry reporting the outcome of 197 BC patients receiving chemotherapy during pregnancy, Loibl et al. found that the median birth weight and the reported infant complications did not differ significantly among the 14 cases exposed in utero to taxanes and those exposed to other cytotoxic agents [17]. Likewise, Cardonick et al. reported that among 15 patients treated with taxanes during pregnancy, there appeared to be no increased risk of fetal or maternal complications compared to other chemotherapy agents [18]. However, more recently, a study of 1170 patients diagnosed with cancer during pregnancy and registered in the International Network on Cancer, Infertility and Pregnancy (INCIP) registry found higher rates of neonatal intensive care unit admissions and SGA neonates among the 84 pregnant patients treated with taxane-containing regimens [5]. Thus, the safe use of taxanes in pregnant patients with cancer remains a subject of controversy.

In this systematic review, data from 321 cases (159 pregnant women and 162 fetuses) were collected to report the obstetric and neonatal outcomes following taxane use during pregnancy. Concerning obstetric outcomes, the incidence of preterm contractions or PROM and pre-eclampsia/HELLP syndrome was similar (11.4% and 5.7%, respectively) to that observed in the general obstetric population (10.0% and 4.6%, respectively) [19, 20]. Similarly, the rates of anemia, thrombocytopenia, and neutropenia were comparable to those occurring in non-pregnant patients treated with taxanes [21]. Notably, although the overall frequency of oligohydramnios/anhydramnios (5.7%) in our review was similar to the rates found in the general obstetric population, we anticipated a lower frequency of events as 72 (54.5%) preterm births were reported. Oligohydramnios/anhydramnios can complicate up to 10% of pregnancies at 40–42 weeks, with pregnancies delivered preterm being significantly less affected (< 1%) [22]. Oligohydramnios/anhydramnios has mainly been reported with the use of trastuzumab, although there is at least one report of a pregnant woman with triple-negative BC who developed it after initiating weekly paclitaxel [23]. Likewise, oligohydramnios has been reported in a pregnant woman with cervical cancer that received platinum-based chemotherapy [24]. As most of the cases of oligohydramnios/anhydramnios (*n* = 4; 67%) reported in this review also received trastuzumab (*n* = 3; 75%) or platinum salts (*n* = 1; 25%), this accounts for the higher rates of oligohydramnios/anhydramnios herein described.

Regarding neonatal outcomes, we found a substantial incidence of preterm delivery and SGA. A total of 93 (70.5%) pregnancies ended preterm, a similar figure to the reported rates of preterm deliveries in pregnant patients with cancer [25–27]. Preterm delivery as an adverse effect of chemotherapy has been extensively reported, both when administered prior to pregnancy and throughout [28, 29]. The mechanisms that associate preterm delivery with chemotherapy are incompletely understood, but might involve placental underdevelopment, reduction of regulatory T cell populations, and apoptosis of fetal membranes [29–31]. It is notable that in our review, only 11.4% of pregnancies were associated with preterm contractions or PROM, which suggests either a heterogenous reporting of preterm contractions/PROM or that most of these preterm births were induced. In regard to SGA, 30.3% of neonates were classified as such. The recent INCIP study reported that amongst chemotherapy drugs, platinum salts were the most closely associated with SGA, with a weaker association for taxanes [5]. The association between chemotherapy, specifically taxane-based regimens, and the risk of SGA remains unclear. In vitro studies suggest that chemotherapy can have a damaging effect on placental tissue, causing reduced placental weight and accumulation of oxidative stress, which could explain the higher observed rates of fetal growth restriction [32, 33]. However, it also likely that other risk factors for impaired fetal growth that are more prevalent in the oncologic population, such as advanced maternal age, malnutrition, and smoking, are at play.

The main perinatal complications reported in this review were not more frequent than in the general neonatal population (20% for hyperbilirubinemia [34] and 5–15% for hypoglycemia [35]). The exception was ARDS, which occurred at a higher frequency (10.6% vs 1.4% in the general neonatal population) [36]. Taxanes can potentially cause lung injury in adult patients through various mechanisms, including interstitial pneumonitis, capillary leakage, and hypersensitivity reactions [37]. Two studies have demonstrated the in vivo transplacental transfer of taxanes, which were consequently detected in newborn meconium [38, 39]. It is plausible that the aspiration of taxane-containing meconium-stained amniotic fluid can increase the risk of ARDS in neonates, but direct evidence supporting this hypothesis is lacking. Additionally, as all neonates with ARDS were born premature, this likely accounts for the high frequency of ARDS. Regarding congenital anomalies, they were relatively infrequent (5.3%) and comparable in occurrence to the global burden of congenital malformations (i.e., 2–3%) [40]. In a study by Cardonick et al., no discernible differences were found in the rates of congenital malformations between the offspring of pregnant patients treated with taxanes and those who were not, although they acknowledge that their sample size may not have been not sufficient to draw firm conclusions [41]. Notably, in said study, the mother of the only neonate with a congenital anomaly (i.e., pyloric stenosis) was treated with multiple anticancer agents, including anthracyclines, cyclophosphamide, and taxanes, which might account for this particular malformation. Interestingly, in our review, among the two neonates exposed to taxanes in the first trimester, none developed a congenital anomaly, which may suggest a low risk of teratogenicity associated with taxanes.

It is important to emphasize that there is limited information regarding the long-term effects of in utero taxane exposure beyond the neonatal period. One study provided a three-year follow-up for eight neonates in whom paclitaxel and its metabolites were detected in meconium [39]. Overall, they reported three birth anomalies: mild hip dysplasia (associated with breech birth), mitral valve stenosis, and cleidocranial dysostosis (an autosomal dominant condition). Other reported health issues included eczema, recurrent otitis media, iron deficiency anemia, upper respiratory infections, and low height and weight, all of which are common comorbidities in the general pediatric population. Thus, it remains unclear if the presence of paclitaxel and its metabolites in meconium holds any clinical significance, especially as their toxic concentration is unknown. The data from our review regarding the health status after birth is reassuring, as most neonates (87%) for whom data was available were reported as healthy at a median follow-up of 16 months. The remaining 13% experienced complications that are not uncommon in the general pediatric population.

Overall, our review suggests that the use of taxanes after the first trimester of pregnancy is safe. There are several biological explanations for this. For instance, there is a decrease in both the area under the curve and maximal plasma concentration of taxanes during pregnancy. Additionally, there is an increase in the distribution volume and clearance of taxanes, which implies a reduced exposure to these drugs in the plasma [42, 43]. Furthermore, reduced placental transfer of taxanes is hypothesized despite their lipophilic properties due to their high molecular weight (> 800 Da for both paclitaxel and docetaxel) and the high placental expression of p-glycoprotein, an efflux transporter [43–46]. Moreover, the expression of p-glycoprotein appears to increase with greater exposure to paclitaxel, leading to an enhanced efflux function and an adaptive function of the human placenta [47]. Additionally, paclitaxel has the potential to upregulate the expression of a transporter associated with platinum efflux, which could theoretically reduce fetal toxicity from taxane-platinum combinations [47]. Finally, although the fetal liver does not yet express CYP3AD (required for the metabolism of both paclitaxel and docetaxel), the maternal liver markedly increases production (by 50–100%) during the third trimester, which may contribute to fetal tolerance to taxanes [41, 48, 49].

In conclusion, administering taxanes during gestation appears to be safe following the first trimester, with maternal and fetal outcomes being similar to the general obstetric and neonatal populations.

## Data Availability

The dataset generated and analysed for the current study is available from the corresponding author on reasonable request.
